# Insecticide-treated bed net use and associated factors among households having under-five children in East Africa: a multilevel binary logistic regression analysis

**DOI:** 10.1186/s12936-022-04416-y

**Published:** 2023-01-07

**Authors:** Tigist Fekadu Seyoum, Zewudu Andualem, Hailemariam Feleke Yalew

**Affiliations:** 1 University of Gondar Comprehensive Specialized Hospital, P. O. Box 196, Gondar, Ethiopia; 2 Department of Environmental and Occupational Health and Safety, Institute of Public Health, College of Medicine and Health Sciences, University of Gondar, P. O. Box 196, Gondar, Ethiopia

**Keywords:** Insecticide-treated bed net, Under-five children, East Africa

## Abstract

**Background:**

Even though malaria is preventable, it remains the leading cause of under-five morbidity and mortality in low-and middle-income countries. Despite the World Health Organization (WHO) recommendations, its advantage, and its free-of-cost access, the utilization of insecticide-treated nets (ITN) is still low in East Africa. Therefore, this study aimed to assess ITN use and associated factors among households having under-five children in East Africa.

**Methods:**

The most recent Demographic and Health Survey (DHS) datasets of East African countries were used. A total of 174,411 weighted samples was used for analysis. Given the hierarchical nature of DHS data, a multilevel binary logistic regression model was fitted to identify factors associated with ITN use. Four models were fitted and a model with the lowest deviance value was chosen as the best-fitted model for the data. Variables with a p-value < 0.2 in the bivariable analysis were considered for the multivariable analysis. In the multivariable multilevel binary logistic regression analysis, the Adjusted Odds Ratio (AOR) with the 95% Confidence Interval (CI) was reported to declare the statistical significance and strength of association.

**Results:**

In this study, the proportion of ITN use among households having under-five children in East Africa was 46.32% (95% CI 46.08%, 46.55%), ranging from 11.8% in Zimbabwe to 70.03% in Rwanda. In the multivariable analysis, being in the age group 25–34 years, married, widowed, and divorced, primary and post-primary education, wealthy households, having a lower household size, many under-five children, having media exposure, and male-headed households were associated with higher odds of ITN use. Moreover, respondents from a rural place of residence, communities with a higher level of media exposure, communities with lower poverty levels, and communities with higher education levels had higher odds of ITN use.

**Conclusion:**

In this study, the proportion of ITN use was relatively low. Both individual and community-level factors were associated with ITN use. Therefore, giving attention, especially to those who had no formal education, the poor, younger age groups, and households with the large family size is advisable to increase awareness about ITN use. Moreover, media campaigns regarding ITN use should be strengthened.

**Supplementary Information:**

The online version contains supplementary material available at 10.1186/s12936-022-04416-y.

## Background

Globally, an estimated 9.7 million under-five children died annually, of them, 41% occurred in sub-Saharan Africa (SSA) [1]. Despite the remarkable progress to achieve sustainable development goals (SDGs) target to reduce the under-five mortality (UFM) rate to 25 per 1000 live births in all countries by 2030, sub-Saharan African countries continue to share the huge burden of the global under-five mortality [2, 3]. Infectious diseases, including malaria, pneumonia, and diarrhoea are the leading preventable causes of under-five mortality specifically in low- and middle-income countries [4, 5]. A wealth of literature reported that identifying contributing factors and working on them is the best option to reverse the huge burden of under-five mortality in SSA [6].

Of the leading infectious causes of morbidity and mortality, malaria remains a major public health issue that affects millions around the world [7, 8]. Malaria is the fourth leading cause of UFM in low-and middle-income countries. As of 2018, an estimated 228 million malaria cases and 405,000 malaria fatalities were reported worldwide [9, 10], of these 93% of malaria cases and 94% of malaria deaths occurred in Africa [11, 12]. Children under 5 years of age and pregnant women are one of the most vulnerable groups affected by malaria [13–15], which is responsible for the death of 285,000 under-five children in Africa [16].

For the effective prevention of malaria among under-five children, the World Health Organization (WHO) recommends the use of insecticide-treated net (ITN), seasonal malaria chemoprevention, intermittent preventive therapy, indoor residual spraying (IRS), and prompt diagnosis and effective treatment of malaria infections [16]. Of these prevention measures, ITN is the core intervention measure identified by the WHO to prevent malaria infection to reduce the incidence of UFM [17]. It is a feasible and cost-effective measure, which decreases child mortality by 17% [18] and new malaria cases by 31% [19]. Besides, it can kill mosquitoes as well as other potential vectors [17, 20].

Despite the WHO recommendations and the advantage of ITN as well as the ITNs free of cost access, its utilization is still low. Globally, the proportion of ITN utilization ranges from 19.5% to 51% [21–23]. According to studies reported in Africa, ITN utilization among under-five children ranged from 11.5% to 51% [21, 22, 24].

Previous studies conducted on ITN utilization found that maternal educational status [25], ITN accessibility and quality [26], respondents age [27], place of residence [28], household size [29], geographic location [23], household wealth status [30], number of under-five children [31], sex of household head [32, 33], access to health information [34, 35], maternal occupation status [25, 36] and distance to reach the health facility were significantly associated with ITN utilization [21, 23, 31, 37–39].

Even though malaria continues as the leading cause of morbidity and mortality in SSA, specifically in East Africa, ITN is underutilized [17, 20] and there is a dearth of information on contributing factors of ITNs utilization among under-five children. Besides, most of the previous studies consider factors at the individual level, and the effects of community-level factors are not assessed very well. The previous studies did not also consider the hierarchical nature of the demographic and health survey data (multilevel analysis), and this might result in a biased estimate.

Therefore, this study was designed with a view of assessing ITN use and its associated individual and community level factors among households having under-five children in East Africa. This study will inform policymakers and other responsible bodies, by identifying the possible factors involved for the utilization of ITN, for effective utilization of ITN to prevent deaths of under-five children due to malaria both at an individual country level and in East Africa in general.

## Methods

### Study area

The most recent DHSs of East African countries were considered for this study. It encompassed East African region which includes 17 countries, of which only 11 countries (Burundi, Tanzania, Kenya, Uganda, Zambia, Zimbabwe, Madagascar, Comoros, Rwanda, Malawi and Mozambique) had complete DHS data regarding ITN utilization.

### Data source, sampling procedure, and population

A secondary data analysis was done based on the DHSs of East African countries, conducted from 2008 to 2019. The DHS survey employs a stratified two-stage sampling technique in each country. In the first stage, Enumeration Areas (EAs) that represent the entire country were randomly selected from the sampling frame (from the available latest national census). The second stage is the systematic sampling of households listed in each cluster or EA and interviews were conducted in selected households with target populations (women aged 15–49 and men aged 15–64). Finally, all households having under-five children in the 11 East African countries were included and the sample size was determined after extracting the pooled data, using *appending* STATA command, based on the availability of the outcome variable in each DHS.

### Variables of the study

#### Outcome variable

ITN utilization was the outcome variable. It was categorized as YES if children under age five were slept under ITN the night before each survey and NO otherwise.

#### Independent variables

Both individual and community level variables were incorporated after searching of literatures.

The independent variables included in this study were: age, marital status of household head, education status of household head, occupation of household head, wealth status, gender of household head, household size, age and gender of the children, number of under-five children**,** media exposure**,** and perception to distance from the health facility. While residence (rural vs urban), level of community poverty, community education level, and level of community media exposure were the community level variables.

### Operational definition

#### Media exposure

It was constructed from three variables: frequency of listening radio, frequency of watching television, and frequency of reading newspaper. Then it was categorized as YES if exposed to at least one of the above media sources and NO otherwise.

#### Community level of education

Measured by the proportion of respondents with a minimum of primary level of education derived from the individual level variable called respondents' level of education. It was classified as low or high based on the national median value as it was skewed. Then, recoded as low (communities in which ≤ 50% respondents had at least primary education) and high (communities in which > 50% respondents had at least primary education) community level of education.

#### Community poverty level

Measured by the proportion of respondents in the poorest and poorer quintiles derived from wealth index. It was classified as low or high based on the national median value as it was skewed. It was coded as “0” for low (communities in which ≤ 50% women had poorest and poorer quintiles), and “1” for higher (communities in which > 50% women had poorest and poorer wealth quintiles) poverty communities.

#### Community level of media exposure

Measured by the proportion of respondents who had exposure to at least one of the above media sources. It was classified as low or high based on the national median value as it was skewed. Then, it was coded/categorized in a similar way to that of the above community level variables as “0” for low and “1” for high community level media exposure (see Additional file 1).

### Data collection tools and procedure

In DHS, five questionnaires were used to collect nationally representative data that reflect the population and health issues relevant to each country every five years. These were: the household questionnaire, the woman’s questionnaire, the man’s questionnaire, the biomarker questionnaire, and the health facility questionnaire. In this study, the household questionnaire to determine the ITN use and associated factors in the region.

### Data quality control

In DHS, pre-test was performed before colleting the data, a debriefing session was held with the pre-test field staff, and adjustments to the questionnaires were made accordingly. Further information regarding the data collection procedure is found in the DHS guide.

### Data processing and analysis

Data of East African countries were appended together using STATA 14 software. Then, recoding and analysis was done, and throughout the analysis, weighting was done to restore the representativeness of the sample so that the total sample looks like the country’s actual population and to get a reliable standard error or statistical estimate.

Descriptive analysis was conducted using cross-tabulations and by calculating frequencies and percentages. To assess the variability of ITN use across communities/clusters, the random effect meausres results such as Intra-class Correlation Coefficient (ICC), the Median Odds Ratio (MOR), and Proportional Change in Variance (PCV) were calculated. To determine the associated factors of ITN use (fixed effects analysis), the multilevel binary logistic regression was used since the data had hierarchical nature. Bi-variable multilevel binary logistic regression was done and these variables with *p*-value < 0.20 in the bi-variable analysis were entered into the multivariable analysis.

While conducting a multilevel analysis, four models were fitted. These were: the null model (a model with no independent variable), model I (adjusted for individual-level variables only), model II (adjusted for community-level variables only, and model III (model adjusted for both individual- and community-level variables simultaneously). Then, to select the best model for the data, the deviance was used. Finally, crude odds ratio (COR) and adjusted odds ratio (AOR) with their 95% confidence interval [40] were calculated for the best-fitted model (a model with both individual and community level variables) and those factors that had *p*-value less than 0.05 in the multivariable analysis were declared to be significant.

## Results

### Individual level characteristics of respondents

A total weighted sample of 174,411 respondents/household heads with under-five children in the household was incorporated in this study. The majority of the mothers of under-five children [99,123 (56.83%)] were married. Around half (49.19%) of the respondents had primary education. Most, 133,925 (76.79%) of the households had one under-five children, whereas only 8818 (5.06%) of the households had three and above under-five children. Regarding media exposure, more than half, 99,061 (56.80%) of the respondents had an exposure to media (radio, television, or newspaper) (Table 1).

**Table 1:** Individual characteristics of respondents

Variables	Weighted frequency (N = 174,411)	Percentage (%)
Age of household head		
11–24 years	14,909	8.55
25–34 years	46,294	26.54
35 and above	113,208	64.91
Marital status		
Single	43,160	24.75
Married	99,123	56.83
Widowed	13,936	7.99
Divorced	18,192	10.43
Educational status of HH		
No education	38,518	22.08
Primary	85,792	49.19
Secondary	37,933	21.75
Higher	12,168	6.98
Wealth status		
Poorest	34,606	19.84
Poorer	34,408	19.73
Middle	33,970	19.48
Richer	35,333	20.26
Richest	36,094	20.69
Household size		
< 5	94,590	54.23
≥ 5	79,821	45.77
Number of under five children		
One	133,925	76.79
Two	31,668	18.16
Three and above	8818	5.06
Sex of HH head		
Male Female	119,972 54,439	68.79 31.21
Media exposure		
No	75,350	43.20
Yes	99,061	56.80
Number of bed nets		
0	58,081	33.30
1	47,397	27.18
2	38,113	21.85
3	20,304	11.64
4	10,516	6.03

### Community level characteristics of respondents

**Fig. 1:**
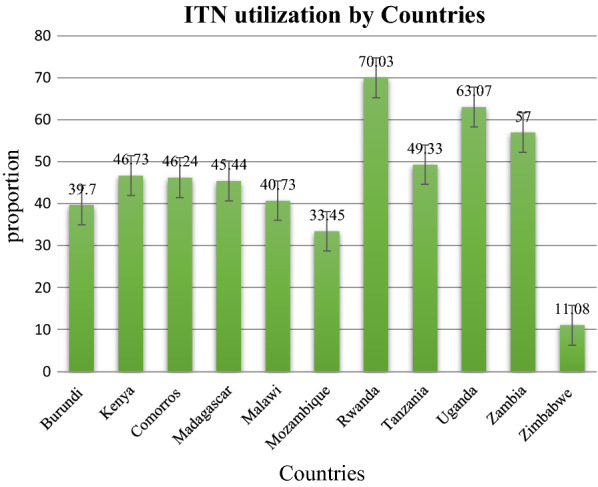
Proportion of ITN utilization by East African region

More than two thirds, 127,310 (72.99%) of the respondents were rural dwellers. More than half of the respondents were from a community with high education level (51.48%), high level of media exposure (52.38%) and high level of community poverty (52.26%). Regarding country, most of the study participants were from Kenya (Table 2; Fig. 1).

**Table 2:** Community level characteristics of respondents

Variables	Weighted frequency (N = 174,411)	Percentage (%)
Place of residence		
Urban	47,101	27.01
Rural	127,310	72.99
Country (with survey year)		
Burundi (2016/17)	15,392	8.82
Kenya (2014)	35,059	20.10
Comoros (2012)	4329	2.48
Madagascar (2008/09)	17,044	9.77
Malawi (2015/16)	25,344	14.53
Mozambique (2015)	13,300	7.63
Rwanda (2019)	12,415	7.12
Tanzania (2015/16)	11,422	6.55
Uganda (2016)	18,409	10.55
Zambia (2018)	11,820	6.78
Zimbabwe (2015)	9877	5.66
Community media exposure level		
Low	83,057	47.62
High	91,354	52.38
Level of community poverty		
High	91,152	52.26
Low	83,259	47.74
Community level of education		
Low	84,629	48.52
High	89,782	51.48

### Proportion of ITN use among households having under-five children in East Africa

The prevalence of ITN use in East Africa was 46.32% (95% CI 46.08%, 46.55%). It had a variation between countries that ranges from 11.8% (95% CI 10.49, 11.72) in Zimbabwe to 70.03% (95% CI 69.22%, 70.83%) in Rwanda (Fig. 1).

### Factors associated with ITN use among under-five children in East Africa

#### Random effects analysis

Table 3 revealed the random effect analysis. As shown from the table, the ICC in the null model revealed that about 15% of the variability of ITN use was attributable to difference between communities or clusters. Besides, the MOR value in the null model revealed that the odds of ITN use was 2.06 times higher among respondents who came from the cluster with higher chance of ITN use as compared to respondents from a cluster with lower chance of ITN use. Moreover, the higher PCV in the final model (Model III) revealed that about 14.4% of the variability of ITN use in East Africa was explained both by individual and community level factors (i.e., model three best explains the variability than other models). The value of all parameters indicates the need to use multilevel logistic regression than ordinary logistic regression. In addition, model III (a model that incorporates both individual and community level factors) had the lowest deviance and hence considered as the best-fit model (Table 3).

**Table 3:** Random effect analysis for the assessment of factors associated with ITN use in East Africa

Parameters	Null model	Model I	Model II	Model III
Community level variance (SE)	0.582 (0.031)	0.552 (0.029)	0.509(0.027)	0.498 (0.027)
ICC	0.150	0.144	0.134	0.131
MOR	2.06	2.03	1.97	1.95
PCV	Reference	0.052	0.125	0.144
Model fitness				
Log likelihood	− 118,231.5	− 116,258.71	− 118,100.78	− 116,182.01
Deviance	236,463	232,517.42	236,201.56	232,364.02

#### Fixed effect analysis

Both individual and community level variables were found to be associated with ITN use. In the multivariable model: age, marital status, education level of the household head, wealth index, household size, number of children in the household, media exposure, gender of household head, residence, community level of media exposure, community poverty level, and community education level were associated with ITN use (*p*-value < 0.05).

The odds of ITN use was 1.10 (AOR = 1.10; 95% CI 1.05, 1.16) times higher among children of households whose head aged from 25 to 34 as compared to those whose household head is aged from 11 to 24 years. Those children of married, widowed, and divorced respondents had 1.42 (AOR = 1.42; 95% CI 1.33, 1.51), 1.24 (AOR = 1.24; 95% CI 1.15, 1.34), and 1.13 (AOR = 1.13; 95% CI 1.05, 1.21) times higher chance of ITN use as compared to single respondents, respectively. The odds of ITN use were 1.23 (AOR = 1.23; 95% CI 1.19, 1.28), 1.20 (AOR = 1.20; 95% CI 1.14, 1.26), and 1.43 (AOR = 1.43; 95% CI 1.33, 1.54) times higher among children of respondents with primary, secondary, and higher education, respectively, as compared to those who had no formal education. Those children from poorer, middle, richer, and richest households had 1.18 (AOR = 1.18; 95% CI 1.13, 1.23), 1.22 (AOR = 1.22; 95% CI 1.16, 1.29), 1.24 (AOR = 1.24; 95% CI 1.16, 1.32), and 1.45 (AOR = 1.45; 95% CI 1.33, 1.57) times higher odds of ITN use, respectively, as compared to those from poorest households. Those children born from respondents with household size of five and above had 3% (AOR = 0.97; 95% CI 0.94, 0.99) lower odds of ITN use as compared to those households with below five household size. Being having two and three and above under five children in the household had 1.25 (AOR = 1.25; 95% CI 1.20, 1.29) and 1.26 (AOR = 1.26; 95% CI 1.19, 1.34) times higher odds of ITN use, respectively, as compared to having one child only. Regarding media exposure, having media exposure was associated with 1.33 (AOR = 1.33; 95% CI 1.29, 1.37) times higher odds of ITN use as compared to their counterparts. When comparing household heads who are female, those male household heads were 4% (AOR = 0.96; 95% CI 0.93, 0.99) times less likely to use ITNs. Moreover, in this study, respondents from rural place of residence (AOR = 1.10; 95% CI 1.02, 1.19), communities with higher level of media exposure level (AOR = 1.20; 95% CI 1.10, 1.32), communities with lower poverty level (AOR = 1.30; 95% CI 1.18, 1.42), and communities with higher level of education (AOR = 1.50; 95% CI 1.37, 1.63) had higher odds of ITN use as compared to their counterparts (Table 4).

**Table 4:** Assessment of factors associated with ITN use among under-five children in East Africa

Variables	ITN utilization		OR (95% CI)	
	Yes (%)	No (%)	COR (95% CI)	AOR (95% CI)
Age of household head				
11–24 years	6622 (44.41)	8287 (55.59)	1.00	1.00
25–34 years	23,068 (49.83)	23,226 (50.17)	1.25 (1.19, 1.31)	1.10 (1.05, 1.16)***
35 and above	51,092 (45.13)	62,116 (54.87)	1.01 (0.97, 1.06)	0.98 (0.93, 1.04)
Marital status				
Single	19,630 (45.48)	23,530(54.52)	1.00	1.00
Married	48,393 (48.82)	50,730 (51.18)	1.37 (1.30, 1.45)	1.42 (1.33, 1.51)***
Widowed	5867 (42.10)	8069 (57.90)	1.02 (0.95, 1.10)	1.24 (1.15, 1.34)***
Divorced	6893 (37.89)	11,298 (62.11)	0.85 (0.80, 0 .91)	1.13 (1.05, 1.21)***
Educational status of HH				
No education	15,017 (38.99)	23,501 (61.01)	1.00	1.00
Primary	40,877 (47.65)	44,915 (52.35)	1.41 (1.36, 1.46)	1.23 (1.19, 1.28) ***
Secondary	18,374 (48.44)	19,559 (51.56)	1.48 (1.41, 1.56)	1.20 (1.14, 1.26)***
Higher	6515 (53.54)	5653 (46.46)	1.86 (1.73, 2.00)	1.43 (1.33, 1.54)***
Wealth status				
Poorest	13,596 (39.29)	21,010 (60.71)	1.00	1.00
Poorer	15,788 (45.88)	18,620 (54.12)	1.29 (1.24, 1.35)	1.18 (1.13, 1.23)***
Middle	16,131 (47.49)	17,839 (52.51)	1.41 (1.34, 1.48)	1.22 (1.16, 1.29)***
Richer	16,786 (47.51)	18,547 (52.49)	1.45 (1.37, 1.54)	1.24 (1.16, 1.32)***
Richest	18,482 (51.20)	17,612 (48.80)	1.77 (1.65, 1.89)	1.45 (1.33, 1.57)***
Household size				
< 5	42,550 (44.98)	52,040 (55.02)	1.00	1.00
≥ 5	38,233 (47.90)	41,588 (51.10)	1.12 (1.09, 1.15)	0.97 (0.94, 0.99)*
Number of under five children				
One	60,175 (44.93)	73,750 (55.07)	1.00	1.00
Two	16,152 (51.00)	15,516 (49.00)	1.30 (1.26, 1.34)	1.25 (1.20, 1.29)***
Three and above	4456 (50.53)	4363 (49.47)	1.26 (1.20, 1.34)	1.26 (1.19, 1.34)***
Media exposure				
No	30,765 (40.83)	44,585 (59.17)	1.00	1.00
Yes	50,017 (50.49)	49,044 (49.51)	1.53 (1.50, 1.56)	1.33 (1.29, 1.37)***
Sex of HH head				
Male	57,758 (48.14)	62,214 (51.86)	1.00	1.00
Female	23,025 (42.29)	31,414 (57.71)	0.77 (0.75, 0.79)	0.96 (0.93, 0.99)*
Place of residence				
Urban	22,410 (47.58)	24,691 (52.42)	1.00	1.00
Rural	58,372 (45.85)	68,938 (54.15)	0.90 (0.84, 0.97)	1.10 (1.02, 1.19)*
Community media exposure level				
Low	38,626 (46.51)	44,431 (53.49)	1.00	1.00
High	42,156 (46.15)	49,198 (53.85)	1.34 (1.24, 1.44)	1.20 (1.10, 1.32)***
Community level of poverty				
High	38,869 (46.68)	44,390 (53.32)	1.00	1.00
Low	41,914 (45.98)	49,238 (54.02)	0.92 (0.85, 0.99)	1.30 (1.18, 1.42)***
Community level of education				
Low	38,099 (45.02)	46,529 (54.98)	1.00	1.00
High	42,683 (47.54)	47,099 (52.46)	1.67 (1.54, 1.80)	1.50 (1.37, 1.63)***

*AOR* Adjusted Odds Ratio, *COR* Crude Odds Ratio

^***^p ≤ 0.001, *p < 0.01

## Discussion

This study aimed to assess ITN use and associated factors among households with under-five children in East Africa. In this study, the proportion of ITN use in East Africa was 46.32% (95% CI 46.08%, 46.55%). This finding is lower than a study conducted in Ethiopia [41] but higher than studies conducted in Ghana [21] and Myanmar [23]. The possible reason for this discrepancy may be due to the sociocultural and socioeconomic differences between countries. The difference between study periods may be the other reason for this discrepancy.

In this study, both individual and community level variables were associated with ITN use. The individual level factors that associated with ITN use were: age of household head, marital status of the household head, educational status of the household head, household wealth status, household size, number of under-five children in the household, media exposure, and sex of household head. Besides, residence, community level of media exposure, community education level, and community poverty level were the community level factors that were associated with ITN use.

The odds of ITN use was higher among under five children whose household head was 25–34 years old as compared to younger age group (aged 11–24 years). This is in agreement with studies conducted in Ghana [21], Kenya [42], and Ethiopia [43]. This could be due to the increased chance of media exposure and frequent use of health facilities by older age groups which increases the use of ITNs for the prevention of malaria for their child [43].

Children whose household head was married, widowed, and divorced had higher odds of sleeping under ITN as compared to single household head. This is in concordance with a study in Ghana [21]. Contrary to a study conducted in Gambia [44], in this study, being having two and three and above under-five children in the household had higher odds of ITN use as compared to having one child only. This could be due to those families with previous history of marital relationship might have advanced age that enables them to get information about ITN use than singles. Similarly, those households with three and above children usually have an increased age than those families with one child. These two reasons might better explain the above associations.

The odds of sleeping under ITN was higher if the household head had primary, secondary, and higher education as compared to those with no formal education. Besides, being from communities with higher education level had higher odds of ITN use. This association of education and ITN use in lines with studies conducted in Gambia, Nigeria, and Guinea [24, 40, 44]. This is because level of education is an important factor in disease prevention as it increases the level of knowledge acquired about different prevention mechanisms of malaria such as ITN use.

Respondents from poorer, middle, richer, and richest households had higher odds of ITN use as compared to those from poorest households. In addition, being from communities with lower poverty level was associated with higher odds of ITN use. This is in agreement with a study conducted in Ethiopia [45]. This might be ascribed to the poor care-seeking behavior among individuals who take care of under-five children belonging to the lowest socio-economic status [46, 47].

In this study, respondents from household size of five and above had lower odds of ITN use as compared to those from households below five household sizes. This is consistent with studies conducted in Ethiopia [29, 43, 48] and Kenya [42]. This could be attributed to the availability of ITNs since households with large household size is difficult to get enough number of ITNs for each members of the household [45, 49].

The study at hand also revealed that, both community and individual level media exposure were associated with ITN use. Having media exposure and being from communities with higher level of media exposure was associated with higher odds of ITN use as compared to their counterparts. This is in agreement with a study conducted in Nigeria [50]. This may be because the distribution of information regarding malaria prevention methods to the individuals and the communities can be achieved with radio, television or newspaper.

In this study, the gender of household head was associated with ITN use. Being from female-headed households was associated with lower odds of ITN use as compared to their counterparts. This is in contrary to a study conducted in Kenya and Sierra Leone [30, 42]. One of the possible explanations for this is that male individuals are better in making decisions about health-related issues, particularly child related ones such as use of ITN and administration of medicines as compared to females. However, the investigator recommends further study in this regard.

Similar to previous studies [23], in this study, children from rural area had higher odds of ITN use as compared to their counterparts. The possible reason for this may be the low incidence of malaria in urban areas, which leads to low perceived threat of mosquito bite.

This study had both strength and limitations. The data used in this study was obtained from nationally representative samples of each East African countries. In addition, it was based on weighting and appropriate statistical analysis (multilevel analysis) to ensure representativeness and to get appropriate statistical estimate. However, since the outcome was assessed based on self-reporting, there may be a possibility of bias where the respondent provides socially acceptable answers. In addition, ITN utilization among under-five children depends on seasons, which was not accounted here. Moreover, some important confounders such as perception of side effects of ITN, knowledge about malaria and its transmission, and environmental factors were not accounted in this study due to lack of variables pertinent to these factors in the DHS data.

## Conclusion

In this study, the proportion of ITN use was relatively low. Both individual and community level factors were associated with ITN use. Being in the older age group, having primary and above educational status, having media exposure, being from rich household, from rural area, being from communities with lower poverty level, being from communities with higher educational level, and being from communities with higher level of media exposure were associated with higher odds of ITN utilization. However, large household size and being from female-headed household were associated with lower odds of ITN use. Therefore, it is advisable to strengthen sharing of information about malaria prevention programme with the different media and consider distribution of ITN based on family size. Besides, special considerations should be given to households with poor socio-economic status and further research, by incorporating important variables such as knowledge and attitude related variables, should be conducted.

## Supplementary Information

**Additional file 1.** Determinant factors of insecticide-treated bed net use among households having under-five children (AOR with 95% CI of all models fitted).

## Data Availability

All result-based data are within the manuscript.

## Abbreviations

AOR: Adjusted odds ratio
DHS: Demographic and Health Surveys
ICC: Intra-class Correlation Coefficient
IRS: Indoor residual spraying
ITN: Insecticide-treated nets
MOR: Median Odds Ratio
PCV: Proportional Change in Variance
SDGs: Sustainable development goals
SSA: Sub-Saharan Africa
UFM: Under-five mortality
VIF: Variance inflation factor
WHO: World Health Organization
