# Supplementary material for: Insecticide-treated bed net use and associated factors among households having under-five children in East Africa: a multilevel binary logistic regression analysis
Source: Malar J. 2023 Jan 7;22:10. doi: 10.1186/s12936-022-04416-y (PMC9826573; doi:10.1186/s12936-022-04416-y)
Supplement: Supplementary file 1 — Additional file 1. Determinant factors of insecticide-treated bed net use among households having under-five children (AOR with 95% CI of all models fitted). [file 12936_2022_4416_MOESM1_ESM.docx]

**Additional file 1. Determinant factors of insecticide-treated bed net use among households having under-five children (AOR with 95% CI of all models fitted)**

|  | Null Model | Model I AOR(95%CI) | Model II AOR(95%CI) | Model III AOR(95%CI) |
| --- | --- | --- | --- | --- |
| Age of household head  11-24 years  25-34 years  35 and above | -  -  - | 1.00  1.11(1.05, 1.16)***  0.99 (0.94, 1.04) | -  -  - | 1.00  1.10 (1.05, 1.16)***  0.98 (0.93, 1.04) |
| Marital status  Single  Married  Widowed  Divorced | -  -  -  - | 1.00  1.41(1.33, 1.50)***  1.22(1.14, 1.32)**  1.12(1.04, 1.20)* | -  -  -  - | 1.00  1.42 (1.33, 1.51)***  1.24 (1.15, 1.34)***  1.13 (1.05, 1.21)*** |
| Educational status of HH  No education  Primary  Secondary  Higher | -  -  -  - | 1.00  1.24(1.19, 1.28) **  1.19(1.13, 1.26)*  1.43 (1.33, 1.54)*** | -  -  -  - | 1.00  1.23 (1.19, 1.28) ***  1.20 (1.14, 1.26)***  1.43 (1.33, 1.54)*** |
| Wealth status  Poorest  Poorer  Middle  Richer  Richest | -  -  -  -  - | 1.00  1.18 (1.13, 1.24)***  1.22 (1.16, 1.28)***  1.21(1.13, 1.28)***  1.36(1.26, 1.47)*** | -  -  -  -  - | 1.00  1.18 (1.13, 1.23)***  1.22 (1.16, 1.29)***  1.24 (1.16, 1.32)***  1.45 (1.33, 1.57)*** |
| Household size  <5  ≥5 | -  - | 1.00  0.97(0.94, 1.00) | -  - | 1.00  0.97 (0.94, 0.99)* |
| Number of under five children  One  Two  Three and above | -  -  - | 1.00  1.25(1.20, 1.29)***  1.26(1.19, 1.33)*** | -  -  - | 1.00  1.25 (1.20, 1.29)***  1.26 (1.19, 1.34)*** |
| Media exposure  No  Yes | -  - | 1.00  1.34(1.30, 1.38)*** | -  - | 1.00  1.33 (1.29, 1.37)*** |
| Sex of HH head  Male  Female | -  - | 1.00  0.96 (0.93, 1.00) | -  - | 1.00  0.96 (0.93, 0.99) |
| Place of residence  Urban  Rural | -  - | -  - | 1.00  0.91 (0.85, 0.97)*** | 1.00  1.10 (1.02, 1.19) |
| Community media exposure level  Low  High | -  - | -  - | 1.00  1.17(1.08, 1.28)*** | 1.00  1.20 (1.10, 1.32)*** |
| Community level of poverty  High  Low | -  - | -  - | 1.00  1.15(1.05, 1.25)*** | 1.00  1.30 (1.18, 1.42)*** |
| Community level of education  Low  High | -  - | -  - | 1.00  1.46(1.35, 1.58)*** | 1.00  1.50 (1.37, 1.63)*** |
